# Exploring the Impact of Neuroticism on Lung Cancer Risk: Insights From Mediated Mendelian Randomization

**DOI:** 10.1002/brb3.70482

**Published:** 2025-04-21

**Authors:** Jie Zhang, Xiao Ma, Zhiyu Liu, He Wang, Binbin Lu, Zhaoxia Wang

**Affiliations:** ^1^ Department of Oncology The Second Affiliated Hospital of Nanjing Medical University Nanjing China; ^2^ Department of Oncology Sir Run Run Hospital Nanjing Medical University Nanjing China

**Keywords:** lung cancer, mendelian randomization, neuroticism

## Abstract

**Objective:**

This study aimed to explore the potential association between neuroticism and lung cancer.

**Methods:**

We conducted analyses on publicly accessible aggregated data from genome‐wide association studies (GWAS) that included individuals of European descent. The objective was to identify single nucleotide polymorphisms (SNPs) significantly associated with neuroticism and utilize them as instrumental variables in a two‐sample Mendelian randomization framework to evaluate the gender‐specific causal link between neuroticism and lung cancer risk.

We applied four statistical methods: Inverse variance weighting (IVW), weighted median, MR‐Egger regression, and weighted mode. Our analysis also considered the mediating effect of educational attainment on this relationship.

**Results:**

We selected 67 SNPs associated with neuroticism at genome‐wide significance levels from GWAS datasets. Our primary findings using IVW suggest a notable increase in lung cancer risk associated with neuroticism across the general population (odds ratio [OR] = 1.175; 95% confidence interval [CI] 1.020–1.354, *p* = 0.026). Gender‐specific analysis revealed that neuroticism posed a slight but significant risk increase in men (OR = 1.006; 95% CI 1.000–1.012, *p* = 0.045) and women (OR = 1.005; 95% CI 1.002–1.009, *p* = 0.002), with findings corroborated by the additional statistical methods. Further, evidence from both observational and Mendelian randomization analyses suggests that genetically predicted neuroticism is causally associated with a modestly increased risk of incident lung cancer, with ∼17% of this effect mediated by educational attainment.

**Conclusions:**

The results from this Mendelian randomization study provide robust evidence supporting a potential association between neuroticism and an increased risk of lung cancer. This association appears more pronounced in men than women. Additionally, educational level serves as a mediator in the nexus between these conditions, suggesting that interventions aimed at increasing educational attainment might mitigate some of the risk neuroticism poses for developing lung cancer.

## Graphical abstract

The results provide robust evidence supporting a potential causal relationship between neuroticism and lung cancer. This association appears more pronounced in men than women. Additionally, educational level serves as a mediator in the nexus. Interventions aimed at increasing educational attainment might mitigate the risk neuroticism poses for developing lung cancer.

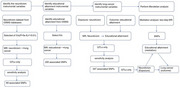

## 1 Introduction

In 2022, nearly 2.5 million new cases of lung cancer were reported, accompanied by over 1.8 million deaths (Siegel et al. 2022). Lung cancer accounts for ∼12.4% of all cancer diagnoses and 18.7% of cancer deaths globally (Schabath and Cote 2019), making it the leading cause of cancer death among men and the second leading cause among women (Torre et al. 2016). In most countries, the 5‐year survival rate for lung cancer remains below 20%. Given its complexity and the interaction between genetic predispositions and environmental factors, identifying risk factors for lung cancer is crucial for developing effective strategies for prevention and treatment.

In recent years, the interconnectedness of mental and physical health has garnered increasing attention in academic discourse (Fava et al. 2016). Scholars assert that personality traits significantly influence health outcomes. Neuroticism, a fundamental personality trait, is characterized by anxiety, emotional instability, and poor self‐awareness, often leading individuals to engage in behaviors that foster negative emotions (Singh 2022). Research indicates that higher levels of neuroticism are linked with compromised mental and physical health and an elevated risk of mortality from all causes (Boyd et al. 2022). Furthermore, conditions such as depression and anxiety disorders are associated with an increased incidence of cancer, reduced cancer survival rates, and higher cancer‐specific mortality (Nakaya et al. 2006). Neuroticism, which encompasses symptoms of both depression and anxiety, shows a particularly strong correlation with lung cancer incidence in some studies, although other research presents conflicting results (Shipley et al. 2007). In one study, there was no association between neuroticism and lung cancer (S. Chen et al. 2024). This inconsistency highlights the need for further investigation of the association and potential mediators that may influence the relationship between neuroticism and lung cancer. Understanding these mediators can help in developing targeted interventions to mitigate the impact of neuroticism on lung cancer risk. Therefore, a new study is warranted.

The rapid advancement of technology has substantially increased the use of genome‐wide association study (GWAS) databases in scientific research (Ma et al. 2024). One promising approach leveraging these resources is Mendelian randomization (MR), a method of causal inference that uses genetic variation as an instrumental variable (IV). This method employs genotypic data as a proxy for random allocation to investigate the association between exposures and outcomes. Specifically, MR, although not fully representative of the trial, could shed light on the role of neuroticism as a potential cause of lung cancer (Larsson et al. 2023). By utilizing two‐sample MR analysis (P. Li et al. 2022), we aimed to explore the association between neuroticism and lung cancer risk and further explore the role of education as a mediator in this association. In addition, no study has reported that neuroticism can be affected by education level and lead to lung cancer.

## 2 Materials and Methods

### 2.1 Mendelian Randomized Study Design

**FIGURE 1:**
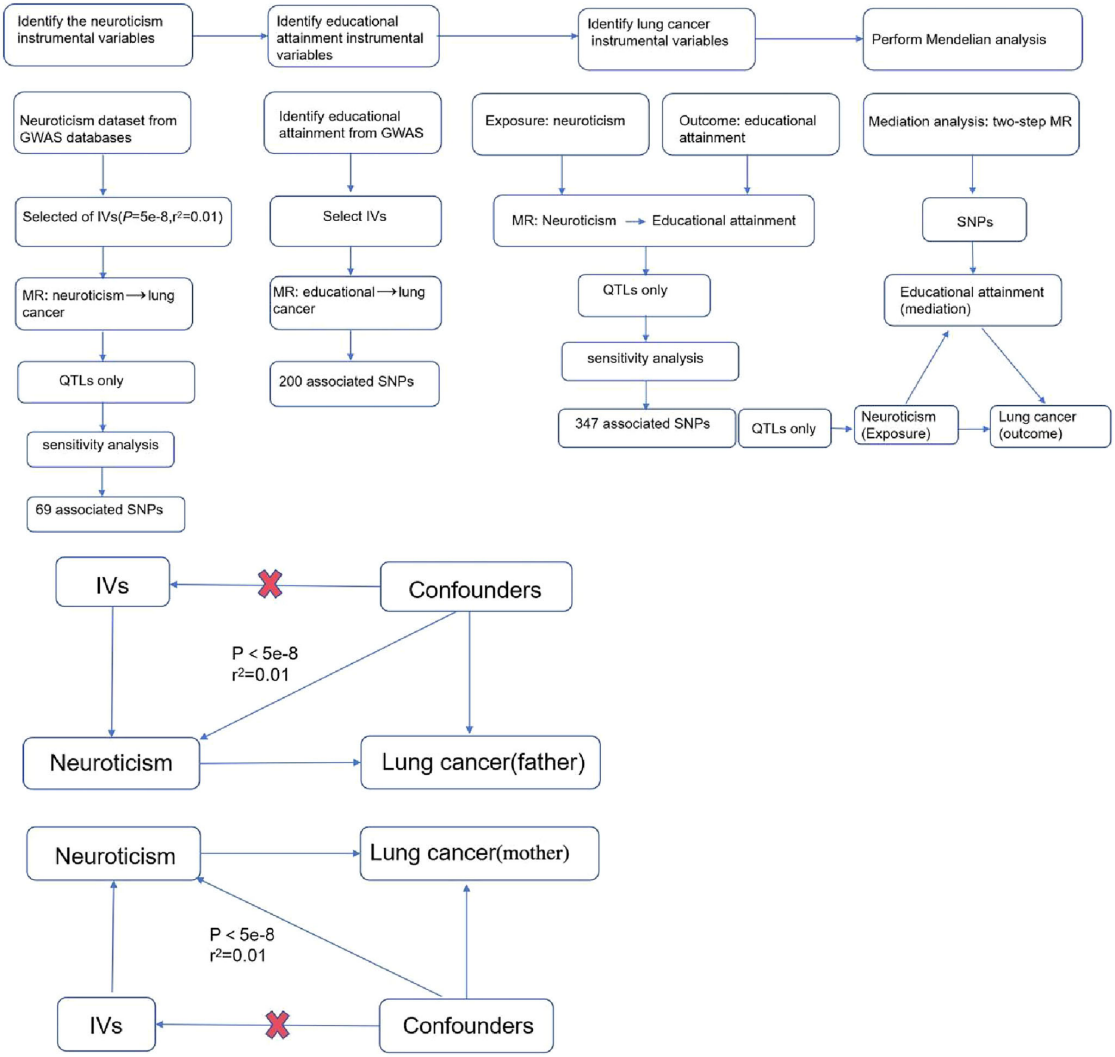
Study flow chart.

MR studies conducted for this research adhered to the STROBE‐MR statement (Skrivankova et al. 2021), employing both two‐sample MR and mediation analysis to enhance methodological rigor. For MR research to yield reliable results, it must satisfy three critical assumptions (H.‐W. Chen et al. 2023): (i) the selected IVs must exhibit a strong association with neuroticism, (ii) the IVs should not be linked to any confounding factors, and (iii) the IVs must influence the outcome solely through the exposure. To ascertain the association between neuroticism and lung cancer, the inverse variance weighted (IVW) method was utilized. Additionally, the reliability of our findings was bolstered by employing the weighted median, MR‐Egger regression, and weighted mode methods. The influence of gender on the relationship between the two diseases was also examined. Building on these analyses, we explored the mediating effects of education level on the relationship between neuroticism and lung cancer. A flow chart outlining the study methodology is presented in Figure 1. The datasets used in this study included neuroticism (ebi‐a‐GCST005232), lung cancer (ebi‐a‐GCST90018875), education (ebi‐a‐GCST90029012), female lung cancer (ukb‐b‐20176), and male lung cancer (ukb‐a‐205). The GWAS of neurosis and lung cancer were from different studies, with a focus on European populations.

### 2.2 Expose and Result Data Sources

After retrieving GWAS data on neuroticism and lung cancer from the IEU OpenGWAS platform, further analyses were conducted using the “TwoSampleMR” package in R software (version 4.2.3). To examine the association between neuroticism and lung cancer, four methods were applied: IVW, weighted median, MR‐Egger regression, and weighted mode. The IVW method served as the primary approach for causal inference. MR‐Egger regression was employed to allow for the inclusion of single nucleotide polymorphisms (SNPs) that may violate the instrumental variable assumptions of Mendelian randomization. The weighted median method, which requires at least 50% valid SNPs to adhere to all three Mendelian randomization assumptions, offers robustness although it has lower statistical power than the IVW method. The relationship between neuroticism and lung cancer was quantified using odds ratios (ORs) and 95% confidence intervals (CIs). Statistical significance was established at a *p*‐value of less than 0.05.

For the exposure variable of neuroticism, we utilized data from a publicly available GWAS meta‐analysis comprising individuals of European descent (*n* = 329,821), which included 18,436,568 SNPs. The lung cancer GWAS dataset was derived from a population sample of European ancestry, encompassing 492,803 individuals, among which were 3791 cases and 489,012 controls. Additionally, the dataset was stratified by gender with 423,258 females and 292,053 males included in the respective lung cancer datasets.

### 2.3 Selected IV

IVs were selected to establish a robust link between major depression and neuroticism. Only IVs with a *p*‐value less than 5 × 10^−8^ were considered statistically significant and chosen for the study (Y. Li et al. 2023). Additionally, IVs demonstrating low linkage disequilibrium (LD) (R^2^ < 0.001) and a substantial physical distance from each other (≥10,000 KB) were selected to minimize bias and potential confounding. An F‐statistic greater than 10 (*F* > 10) was employed to further exclude bias associated with weak instrumental variables. We use GWAS Catalog website (https://www.ebi.ac.uk/gwas/) (Sollis et al. 2023) to IVs review, in order to identify and eliminate any potential confounding factors related factors. SNPs found to be associated with both outcomes and confounders were excluded from the analysis.

### 2.4 Mediation Analysis

We examined the role of educational attainment on gender‐specific neuroticism in the development of lung cancer. Our analysis utilized the intermediary database sourced from the ebi‐a‐GCST90029012 dataset, which comprises 470,941 individuals and 11,972,619 SNPs. The IVW method served as our primary approach to evaluate the relationship between neuroticism, educational attainment, and lung cancer. We focused on distinguishing the direct effect of neuroticism on lung cancer (denoted as b3) (Wang et al. 2023). The indirect effect, mediated by educational level, was determined using the product of coefficients method (b1 × b2). To assess the proportion of the total effect mediated, the indirect effect b1 × b2 was divided by the direct effect b3. Confidence intervals for the indirect effects were calculated using the delta method.

### 2.5 Heterogeneity and Sensitivity Analysis

Heterogeneity was assessed using the Cochran *Q* and *I* ^2^ tests. To evaluate the sensitivity of the overall results, we employed the leave‐one‐out method, wherein each study was sequentially excluded to determine its influence on the collective outcomes. Additionally, the robustness of the remaining instrumental variables was tested by individually removing a specific SNP and recalculating the power of the instrumental variables.

### 2.6 Statistical Analysis

All analyses were conducted using the R software (Version 4.2.3), specifically employing the “TwoSampleMR” and “MR‐PRESSO” packages. A *p*‐value above 0.05 was considered indicative of no statistically significant association between the exposure and outcomes.

## 3 Result

### 3.1 SNP Details

Detailed Mendelian randomization results for both diseases and mediators can be found in Supporting Information. Sixty‐seven SNPs demonstrated a significant positive association with neuroticism. Estimates for each SNP's association with neuroticism and lung cancer—including beta values, standard error (SE), OR value, and *p*‐values—are provided.

### 3.2 Two‐sample MR

Neuroticism was found to be genetically associated with an increased overall risk of lung cancer, with an OR of 1.175 (95% CI: 1.020–1.354; *p* = 0.026). For gender‐specific analyses, the OR for males was 1.006 (95% CI: 1.000–1.012; *p* = 0.045) and for females was 1.005 (95% CI: 1.002–1.009; *p* = 0.002), indicating a significant association in both groups. After excluding SNP‐related confounders using the gwas catalog search, it was found that there was no neuroticism‐related factor affecting the outcome (Table 1).

### 3.3 Intermediate MR

Mediation analysis revealed that educational level mediated the relationship between neuroticism and the overall risk of lung cancer. Specifically, neuroticism was negatively associated with the likelihood of completing college, indicated by an OR of 0.959 (95% CI: 0.952–0.967, *p* = 2.47E‐23). Additionally, educational level was inversely associated with lung cancer risk, with individuals holding a university degree or higher exhibiting a significantly lower risk compared to those without a university degree (OR: 0.504, 95% CI: 0.330–0.770, *p* = 0.002). The mediating effects are detailed in Table 1 Table 2.

**TABLE 1:** Each method of Mendelian randomization assessed the causal effect of neuroticism on lung cancer risk.

Method	nsnp	Beta	SE	pval	OR	Low95%	Up95%
MR Egger	67	0.146	0.534	0.140	1.157	0.406	3.296
Weighted median	67	0.106	0.100	0.037	1.112	0.913	1.354
Inverse variance weighted	67	0.161	0.072	0.019	1.175	1.020	1.354
Weighted mode	67	0.073	0.198	0.030	1.076	0.730	1.585

**TABLE 2:** The mediating effect of neuroticism on the increased risk of lung cancer was assessed by educational level.

Mediator	Total effect b3	Direct effect b1	Direct effect b2	Mediation effect	Mediated proportion (%)
Educational level	0.161	−0.041	−0.685	0.174	17.4

### 3.4 Heterogeneity and Sensitivity

The Cochran *Q* and *I* ^2^ tests revealed no significant heterogeneity among the studied SNPs, suggesting consistent causal estimates across SNPs as detailed in Supporting Information 2. Further analysis indicates that no individual SNP has a decisive impact on causal inference. Additionally, the funnel plot demonstrated the absence of outliers in these results. Moreover, there was no evidence of pleiotropy affecting the outcomes, as indicated by the MR‐Egger test (*p* > 0.05).

## 4 Discussion

The possible influence of personality dynamics on cancer formation, progression, and eventual outcome has been the subject of heated debate (Blanchard and Abell 2019; Jokela et al. 2014). Previous studies have exhibited considerable variation in the psychiatric disorders examined and the specific cancer endpoints of interest (Grassi and Riba 2020). Employing clear and precise definitions of mental illnesses is crucial due to their potential to induce diverse physiological and behavioral effects. Research has established a genetic linkage between major psychiatric conditions, including neuroticism, and both smoking and lung cancer (Wei et al. 2022). However, causation is not likely to be mediated solely by smoking, indicating the necessity to explore other potential mediators. In this two‐sample MR study with mediation analysis, we observed that neuroticism was associated with an elevated risk of lung cancer in the general population. Notably, the incidence of lung cancer was significantly higher in men compared to women. To further dissect this relationship, we stratified the data by sex and investigated the causal links between neuroticism and lung cancer in both genders. Our findings indicate that neuroticism correlates with lung cancer risk in both men and women, with neurotic men exhibiting a particularly higher risk.

We subsequently performed an intermediate MR analysis to explore the potential mediating role of educational attainment in the relationship between neuroticism and lung cancer risk. After adjusting for educational attainment in the MR framework, the previously observed association between neuroticism and lung cancer risk diminished. This finding suggests that educational attainment may mediate this relationship. The mediational analysis indicated that educational attainment accounted for part of the association between neuroticism and the overall risk of lung cancer. The study also revealed a lower risk of lung cancer among individuals who had earned a bachelor's degree, providing a fresh perspective for future research on underlying mechanisms.

The putative role of personality in cancer risk is controversial, and the evidence is inconclusive (Huggan 1968). Some studies have shown that there is no obvious association between personality traits and lung cancer (S. Chen et al. 2024). However, in a meta‐analysis (Jokela et al. 2014), personality traits were all associated with the incidence of six specific cancers, including lung cancer, and were not associated with cancer mortality. In a 16‐year follow‐up study, adversarial personality traits were associated with an increased risk for smoking‐related cancer (Lemogne et al. 2013). Consistent with our results, multiple studies have demonstrated a link between education and lung cancer risk. According to the 2021 US Health Survey (Korn et al. 2023), among lung cancer patients aged 45 years or older, the highest prevalence (8.6%) was noted in those with educational attainment below high school. Conversely, lung cancer rates decreased with higher education levels, exhibiting the lowest prevalence (1.6%) among individuals holding a bachelor's degree or higher. Similarly, a large Norwegian cohort study (Larsen et al. 2020) found that irrespective of smoking status, men and women with lower educational levels were more likely to die from lung cancer compared to those with higher educational achievements. Furthermore, a Mendelian randomization study on education and lung cancer (Zhou et al. 2019) identified low educational attainment as a risk factor for the development of lung cancer. Although smoking remains a primary risk factor, accounting for 90% of lung cancer cases, the indirect effects of smoking differ by educational level, with the most pronounced effects observed among those with the lowest education. Comparing elderly cancer patients with those without cancer, the prevalence of high neuroticism was similar. High neuroticism was confirmed to be associated with negative health and lifestyle problems in both groups (Grov and Dahl 2021; Kissen and Eysenck 1962). However, in cancer patients, low levels of neuroticism and high levels of extraversion personality traits are associated with mental health of patients (Macía et al. 2020).

This MR study presents several advantages (Birney 2022). First, it used aggregated GWAS data on neuroticism and overall lung cancer risk from European populations to minimize potential bias. Second, the study not only established the association between neuroticism and lung cancer risk but also explored this association by sex, further investigating how education mediates this relationship. However, there are notable limitations to this MR study (Yuan and Larsson 2022). Primarily, as the study predominantly included a European cohort, the generalizability of the association between neuroticism and lung cancer risk across different ethnic populations remains uncertain. Additionally, the genetic association between neuroticism and lung cancer risk identified in this study was not corroborated by secondary databases, which could affect the robustness of the findings. Lastly, the study did not conduct further stratifications by tumor type and disease stage, which could provide deeper insights into the relationship.

## 5 Conclusion

In conclusion, our mediated MR study demonstrates the association between neuroticism and an increased overall risk of lung cancer, affecting both male and female groups. Educational attainment plays a significant role as a mediator in this association. Consequently, individuals with neurotic personalities may require increased clinical surveillance for lung cancer, facilitating early detection and potentially improving outcomes.

## Author Contributions

**Jie Zhang**: Data curation, supervision, writing ‐ original draft. **Xiao Ma**: Conceptualization, formal analysis, writing ‐ review and editing. **Zhiyu Liu**: Methodology, resources, visualization. **He Wang**: Resources, software, investigation. **Binbin Lu**: Data curation, project administration, funding acquisition. **Zhaoxia Wang**: Writing ‐ review and editing, conceptualization, validation.

## Conflicts of Interest

The authors declare no conflicts of interest.

### Peer Review

The peer review history for this article is available at https://publons.com/publon/10.1002/brb3.70482.

## Supporting information



Supplementary Materials.

Supplementary Materials.

## Data Availability

The data that support the findings of this study are available in the Supporting Information of this article.
