# Supplementary figures and images for: Exploring the Impact of Neuroticism on Lung Cancer Risk: Insights From Mediated Mendelian Randomization
Source: Brain Behav. 2025 Apr 21;15(4):e70482. doi: 10.1002/brb3.70482 (PMC12012258; doi:10.1002/brb3.70482)

1、ebi-a-GCST005232-ebi-a-GCST90018875


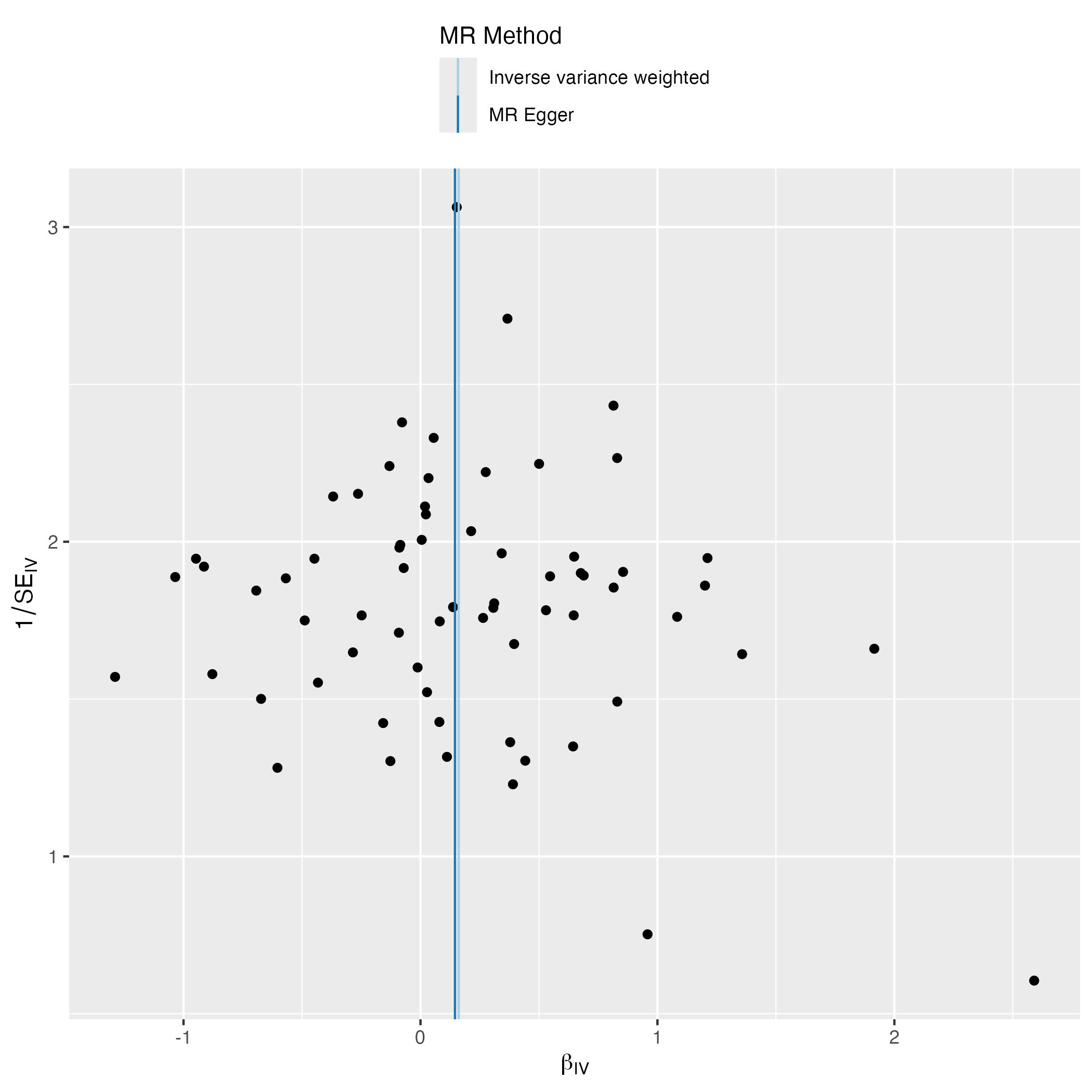

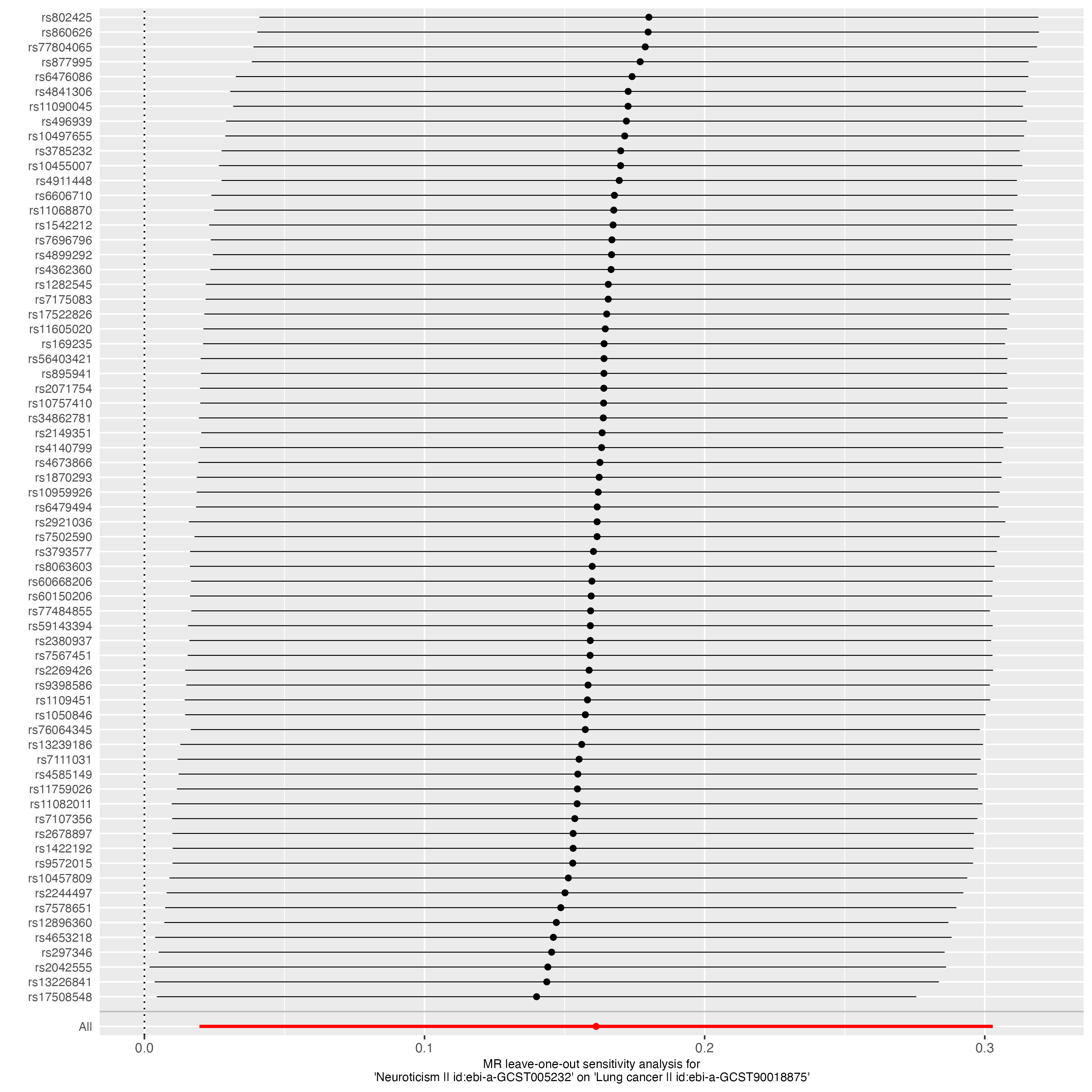

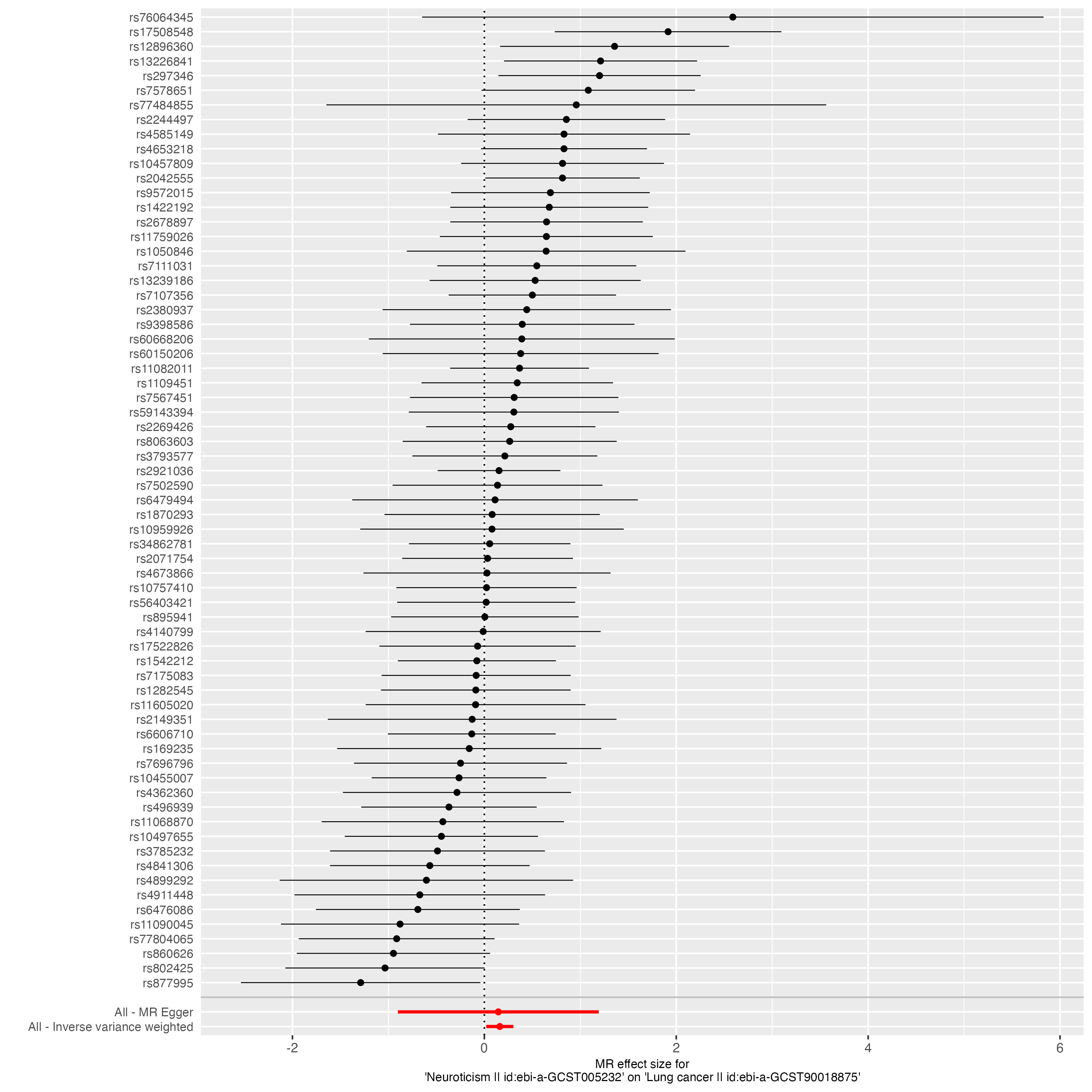


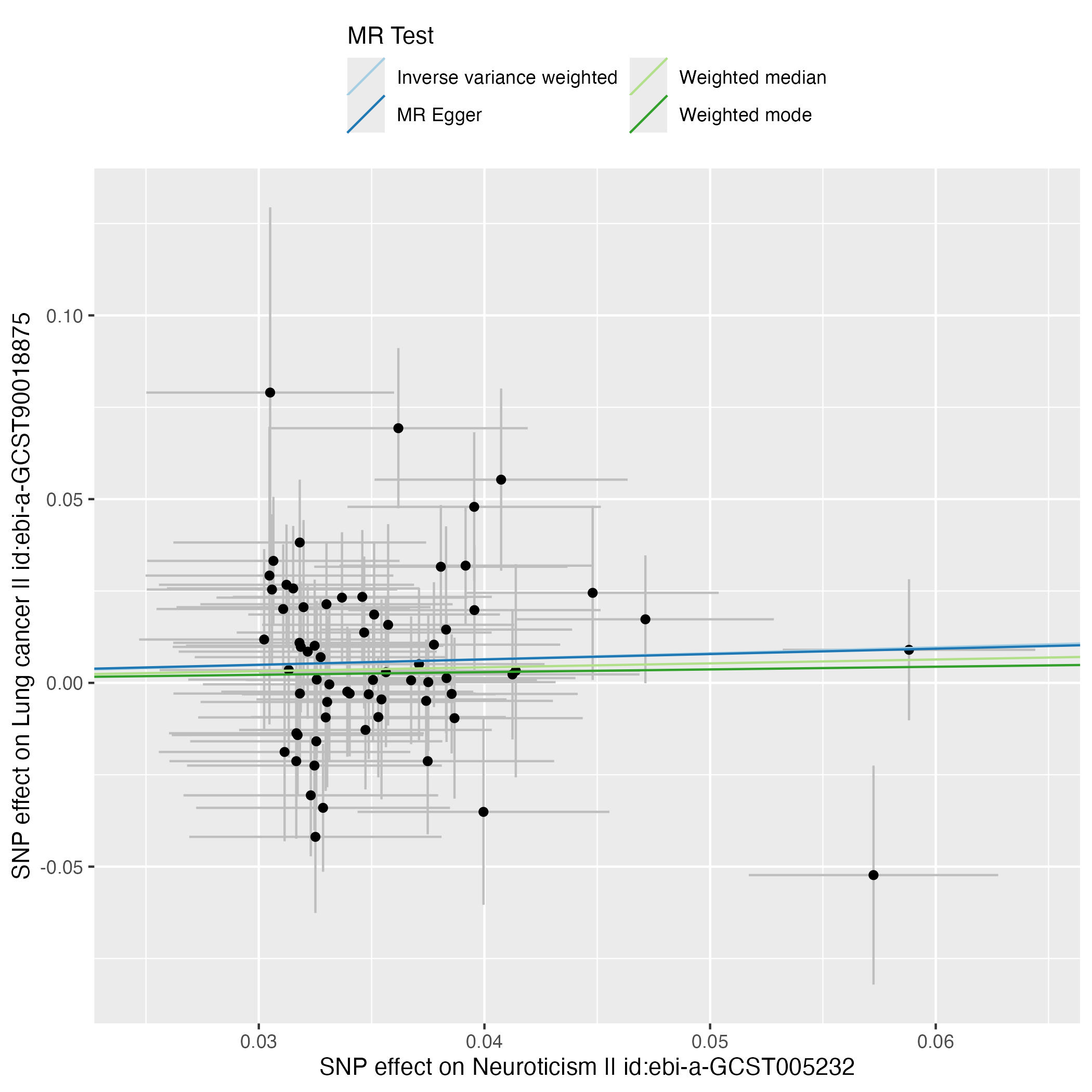


2、ebi-a-GCST005232-ukb-b-20176


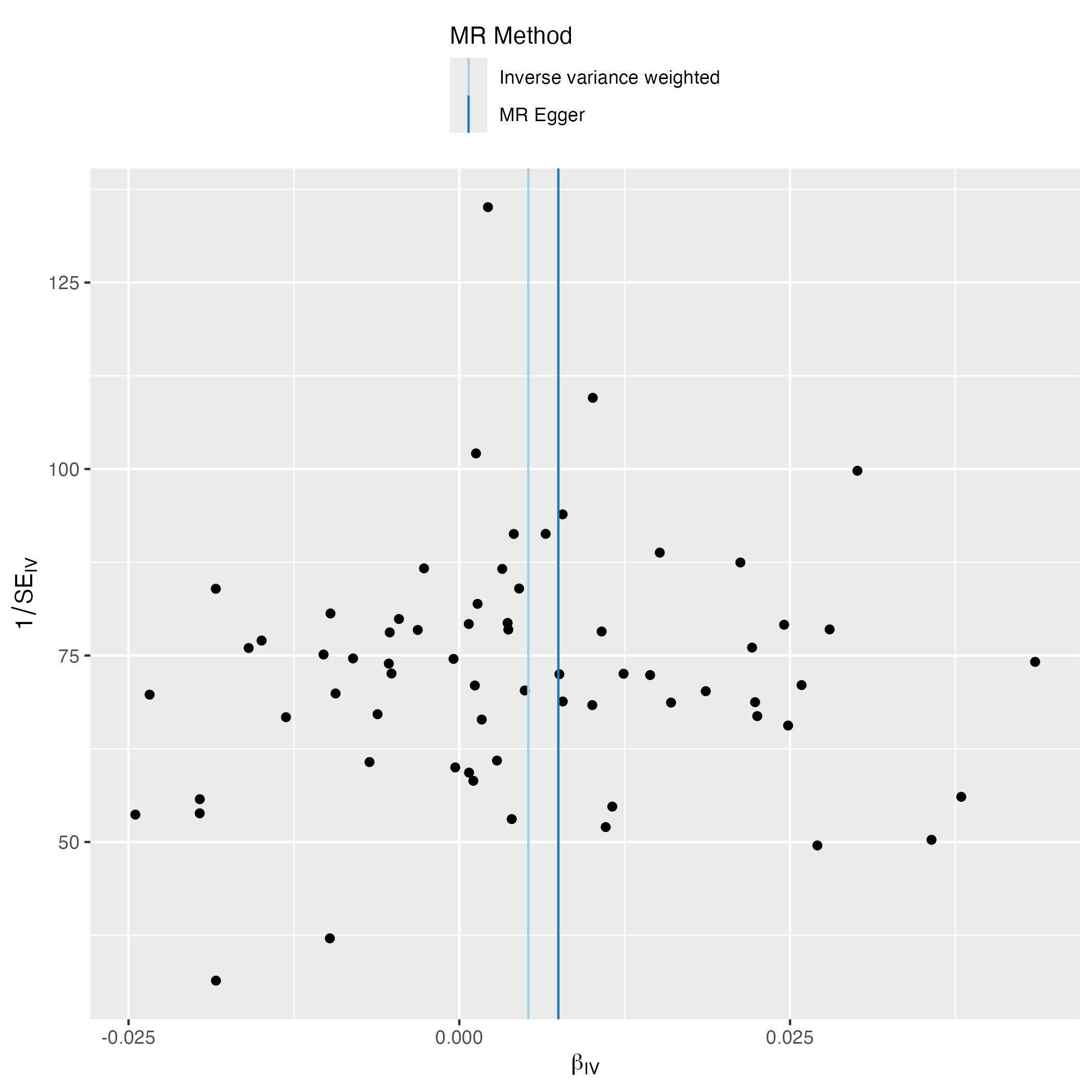

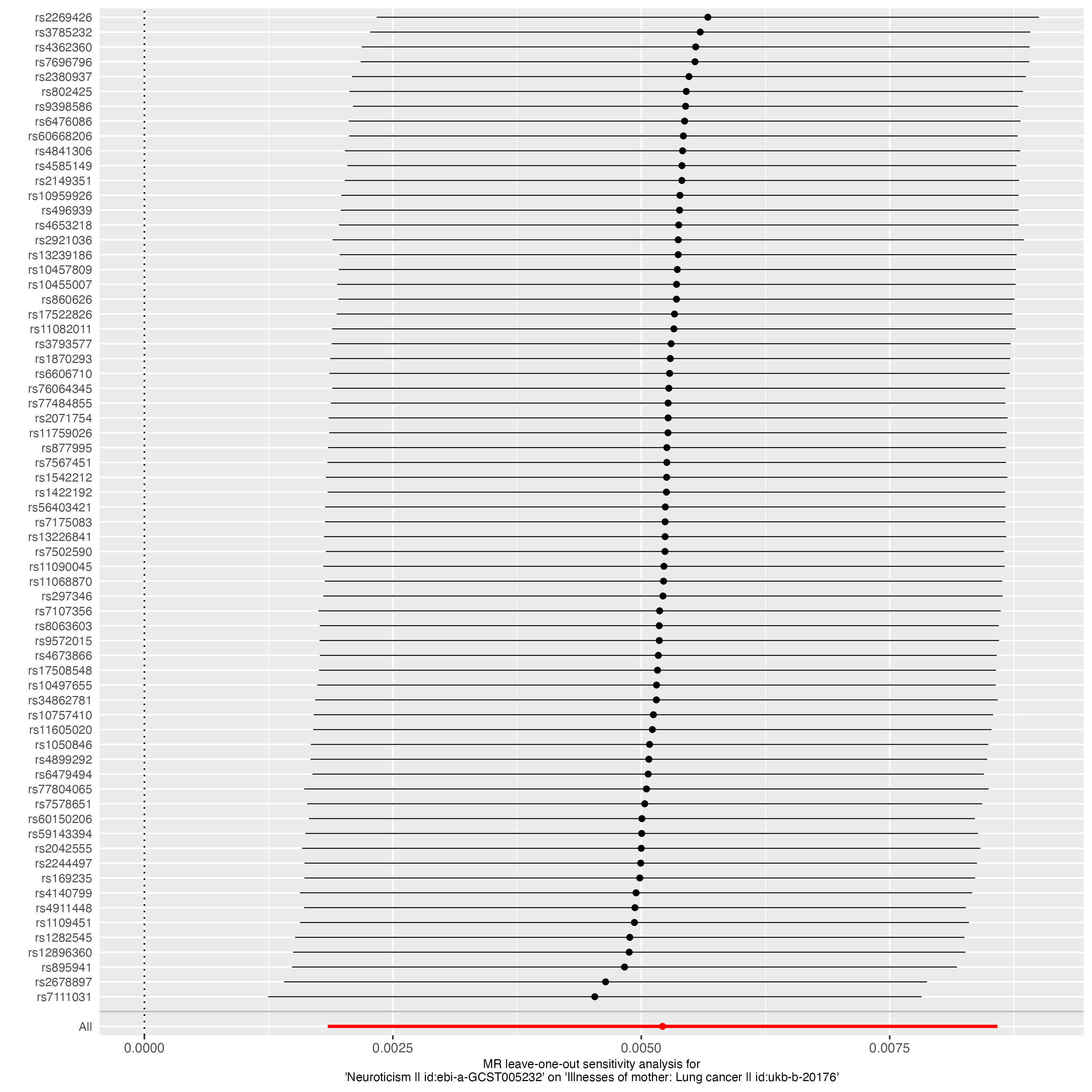

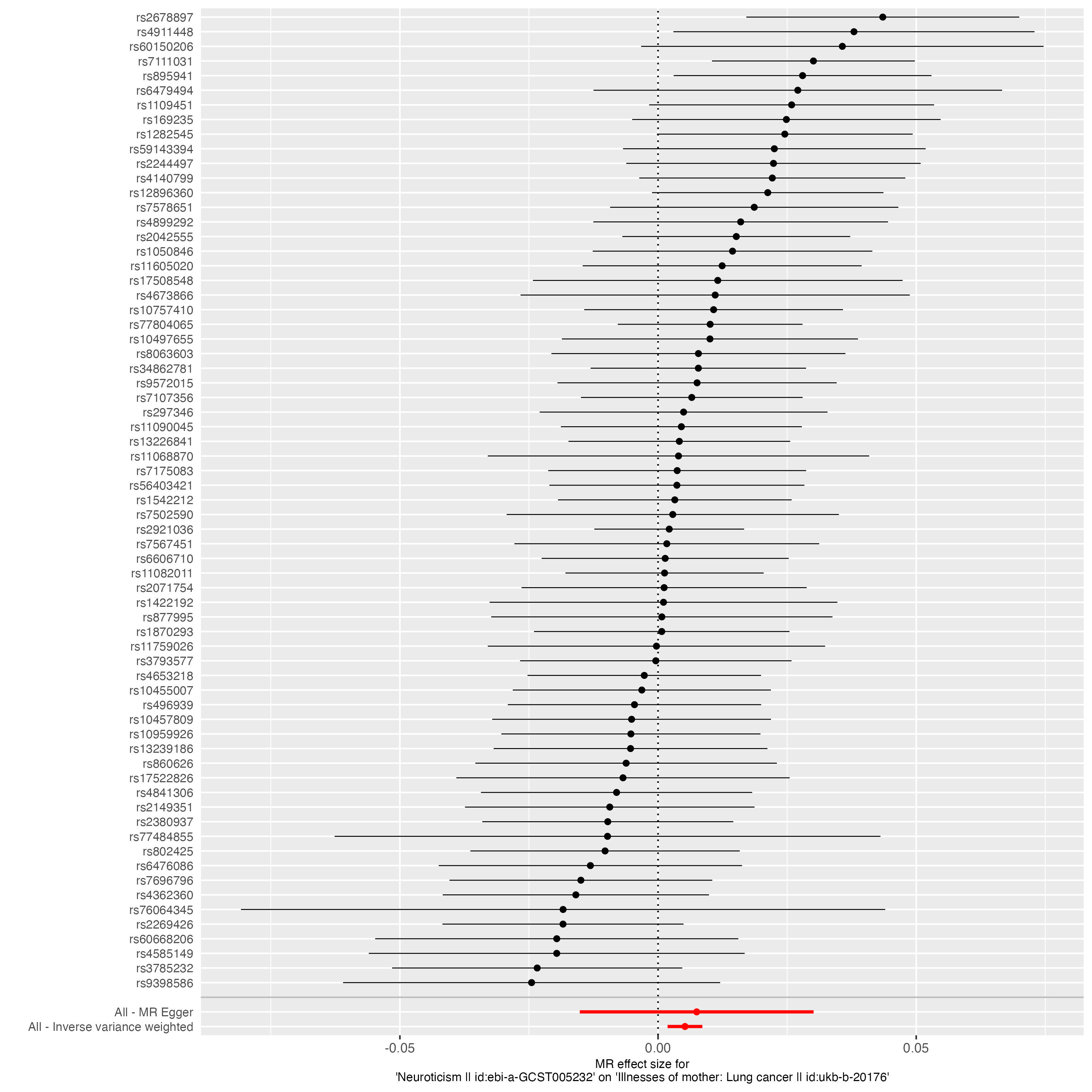

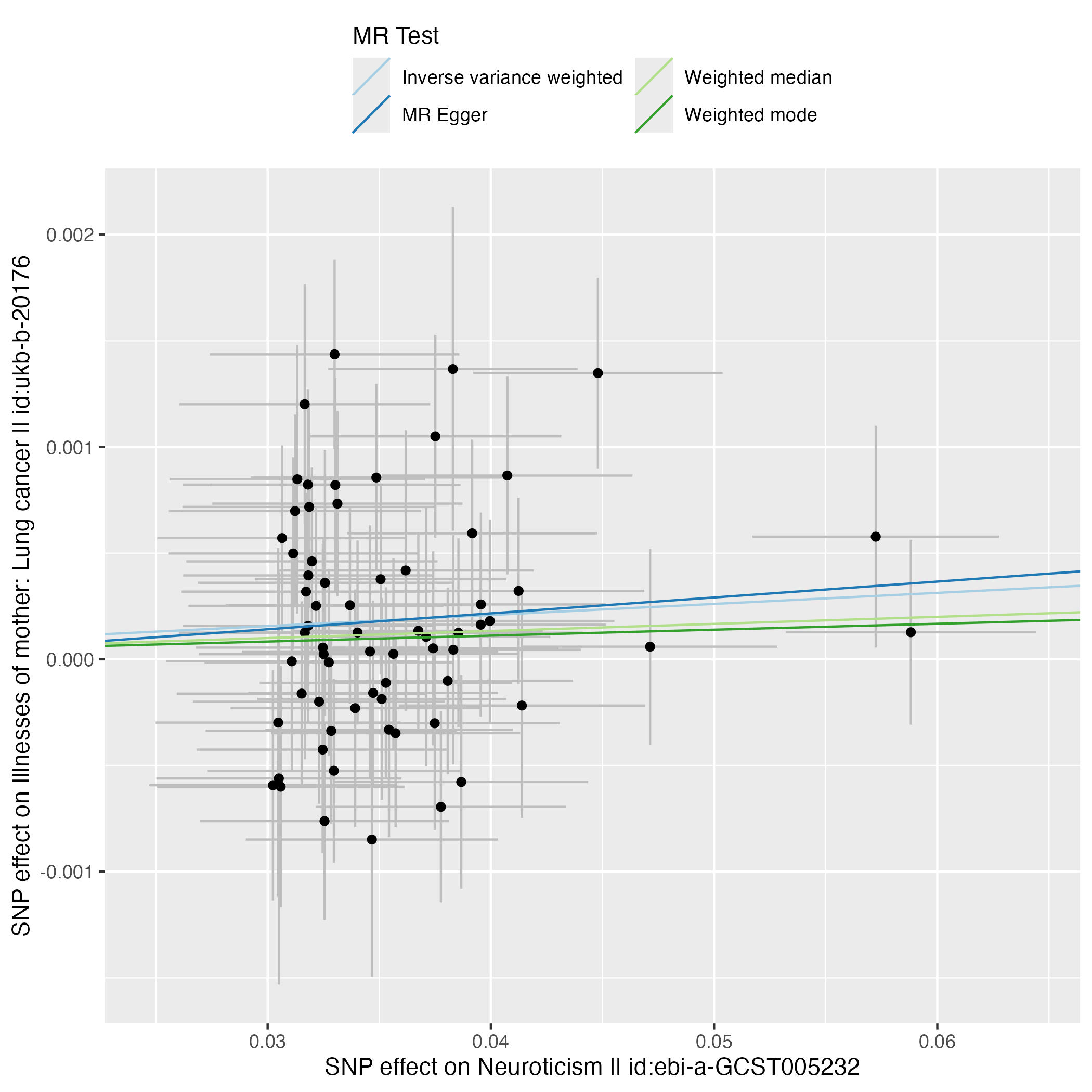


1. ebi-a-GCST005232-ukb-a-205


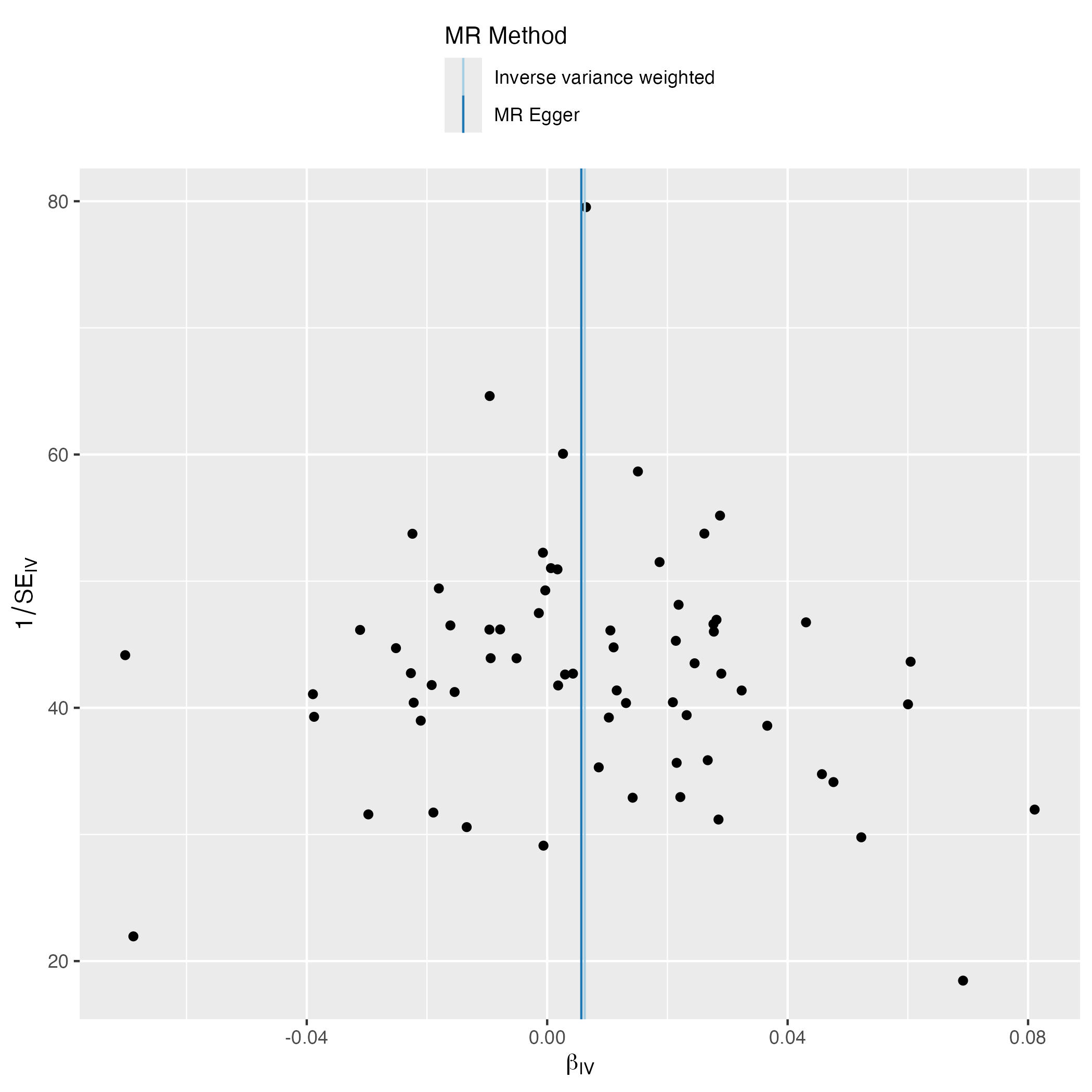

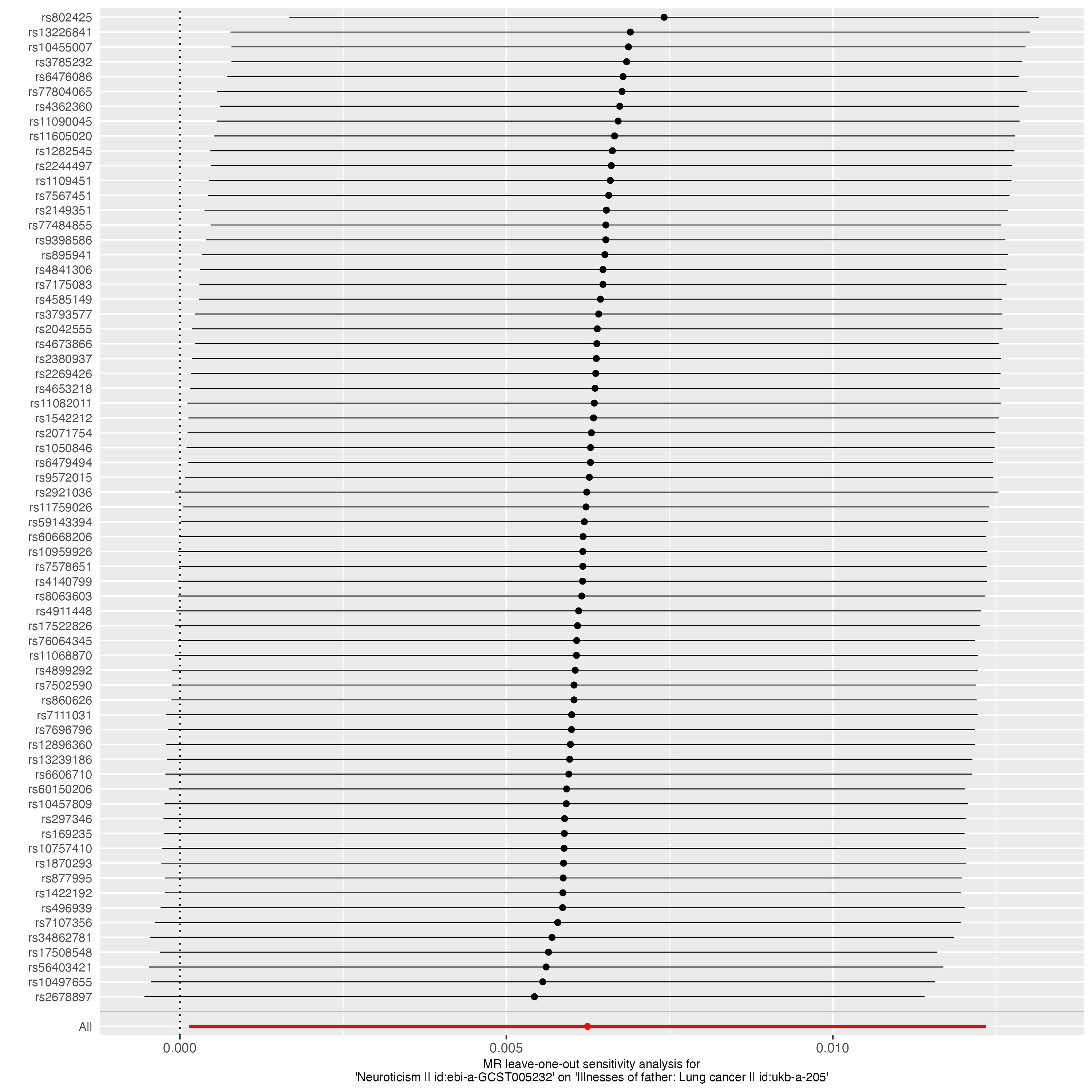

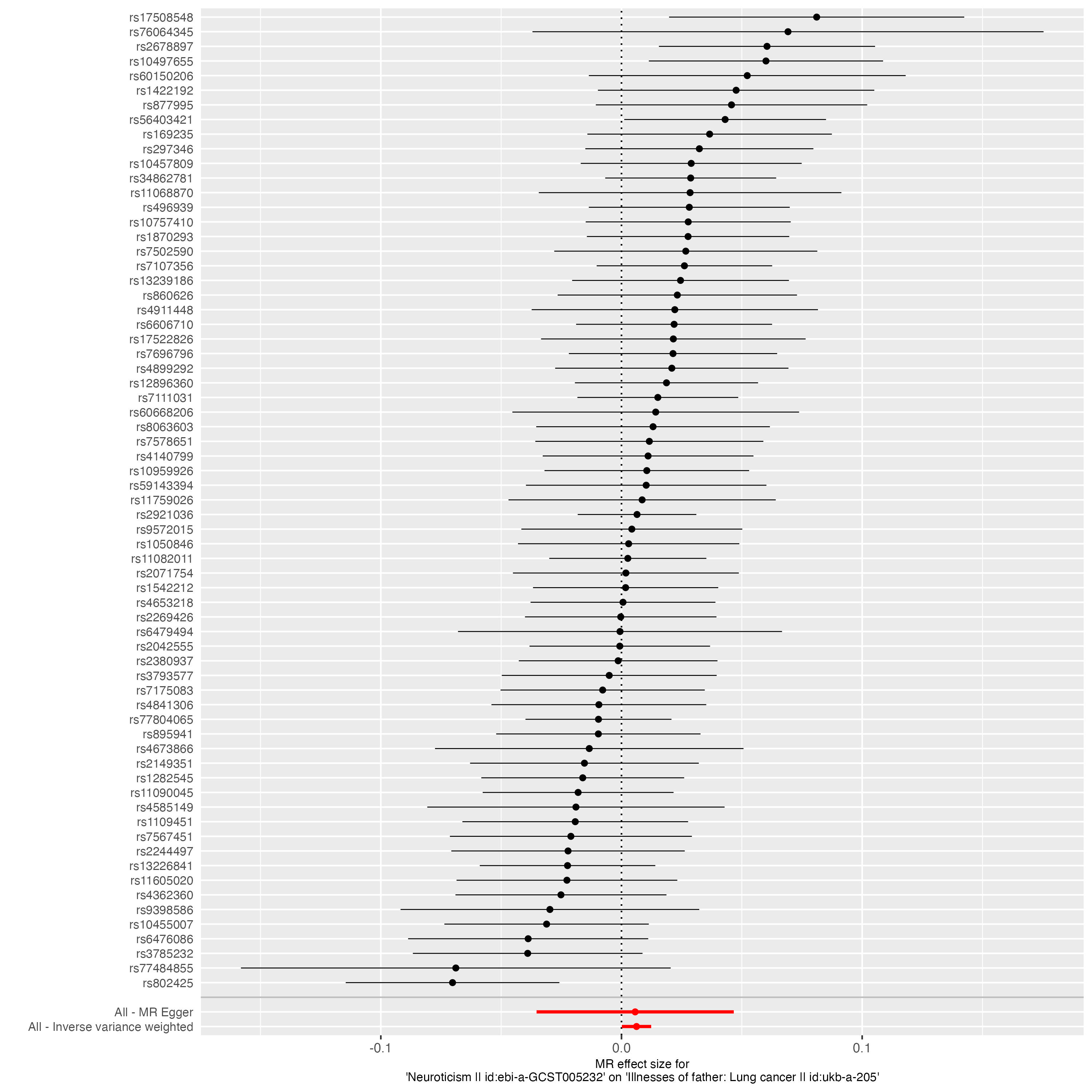

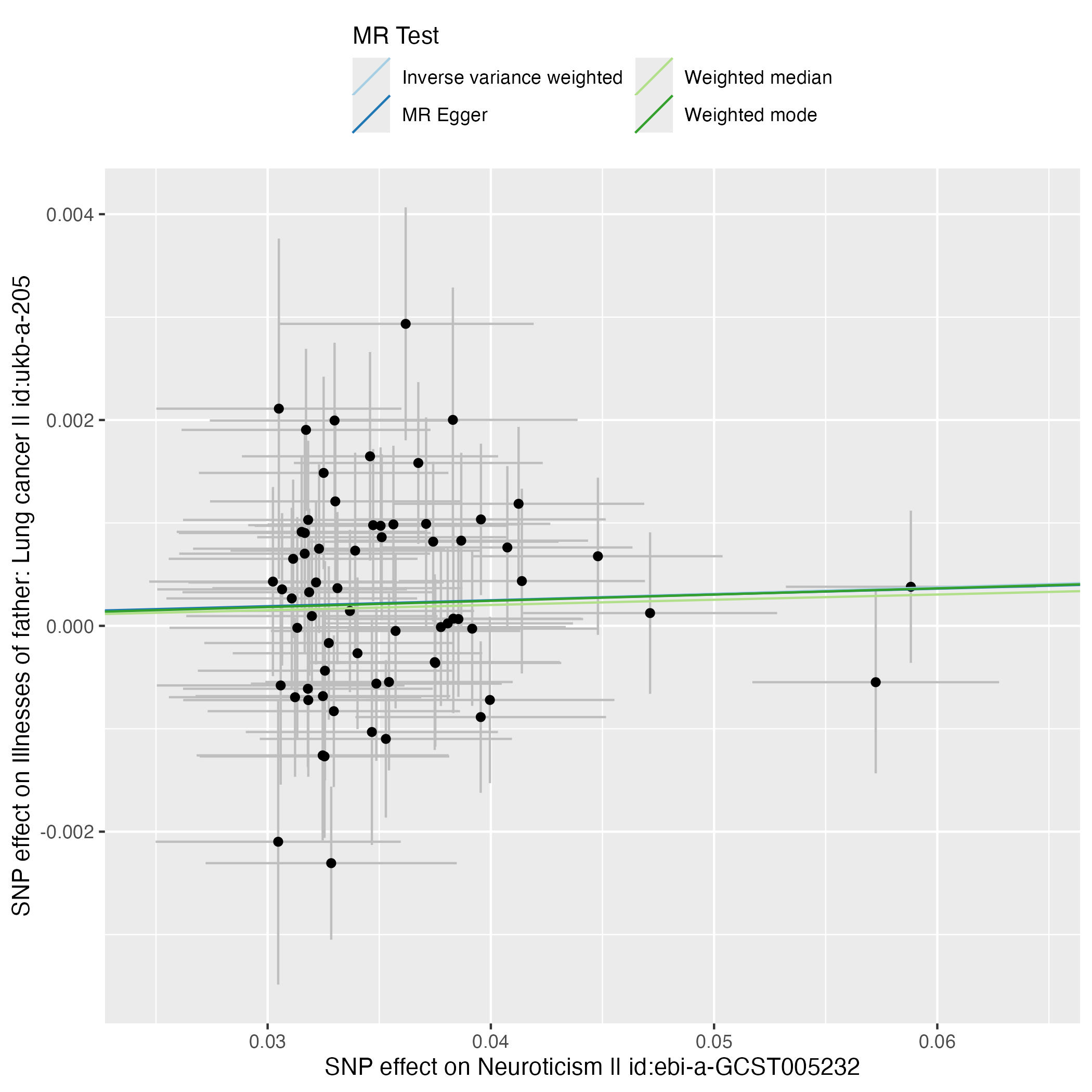

Supplement: Supplementary file 2 — Supplementary Materials. [file BRB3-15-e70482-s001.docx]
